# Postnatal Depression Beyond 12 Months: A Systematic Review and Meta‐Analysis

**DOI:** 10.1111/inm.70018

**Published:** 2025-03-07

**Authors:** Elsie Hellyer, Katrina Nash, Ellie Jones, Alice Sitch, Jelena Jankovic, Giles Berrisford, Amelia Casey, Christine MacArthur

**Affiliations:** ^1^ Aintree University Hospital NHS Foundation Trust Liverpool UK; ^2^ Oxford University Clinical Academic Graduate School Oxford UK; ^3^ Department of Applied Health Sciences University of Birmingham Birmingham UK; ^4^ NIHR Birmingham Biomedical Research Centre University Hospitals Birmingham NHS Foundation Trust and University of Birmingham Birmingham UK; ^5^ Birmingham and Solihull Mental Health NHS Foundation Trust Perinatal Mental Health Service Birmingham UK

**Keywords:** postnatal care, postnatal depression, prevalence, systematic review

## Abstract

Traditionally, postnatal depression (PND) has been considered as depression in the first year after giving birth, although it has been argued that the 12‐month cut‐off may be somewhat arbitrary. Specialist perinatal mental health services in England have recently been extended to include women in their second year postpartum; however, there is no good estimate for the prevalence of PND beyond the first year. This review aimed to obtain the best estimate of the prevalence of PND in the second postpartum year. Eligible studies were those that assessed PND and provided a point prevalence using a validated screening tool or clinical diagnosis at least once beyond the first 12 months in women over the age of 18 years in any country. Studies were excluded if they only included women who were already depressed or had elevated depression scores at baseline. PubMed, Embase, Web of Science, CINAHL and PsychINFO were searched in January 2021 (and updated in February 2024) for studies that included the prevalence of PND beyond the first 12 postnatal months. Study quality was assessed using Cochrane's ROBINS‐I and Risk of Bias 2 tools. Prevalence data were combined in meta‐analysis using prediction intervals (PIs). A total of 6340 papers were found, and of these, 32 studies including 57210 participants across 18 countries met the inclusion criteria and were meta‐analysed. The prevalence of PND in the second year (13–24 months) was 15% (95% confidence interval [CI] 12%, 17%; 95% PI 4%, 30%) and similar to that in the first year, 16% (95% CI 14%, 19%; 95% PI 6%, 31%). Despite considerable heterogeneity, common in meta‐analysis of prevalence studies, findings show that a similar proportion of women experience PND in the second year after birth.

## 1 Background

Postnatal depression (PND) is generally defined as depression experienced in the first 12 months postpartum and is the most common perinatal mental illness globally, affecting 10%–15% of women having a baby (National Institute for Health and Clinical Excellence 2014; World Health Organisation 2008). Symptoms are similar to those in depression at other times and include low mood and markedly diminished interest or pleasure in all or most activities (World Health Organisation 1993). It can be accompanied by multiple somatic symptoms such as difficulty sleeping, changes in appetite and concentration, and negative cognitions, often about one's worth or value as a mother (Howard et al. 2014; World Health Organisation 1993). PND can have a major impact on the mother, child and family, continuing in the longer term (Hakanen et al. 2019; Brummelte and Galea 2016), and has a major economic and social long‐term cost (Bauer et al. 2014). A higher prevalence of PND is reported in women in low‐ and middle‐income countries compared to that in women in high‐income countries (Parsons et al. 2012).

There is overwhelming evidence that specialist (psychiatric and psychological) perinatal mental health (PMH) services, which are also provided during pregnancy, are necessary to improve outcomes for women with perinatal mental illness (including PND) and their babies (National Collaborating Centre for Mental Health 2018; HM Government 2021). This is because there are differences in context when treating women during this time, and PND can have wide‐ranging negative effects on child development, including impairments in cognitive performance, behaviour disturbance and insecure attachment, which can persist into late childhood and adolescence (Stein et al. 2014; Howard et al. 2018). Psychological and psychosocial interventions are effective treatments for PND (Howard et al. 2014; Dennis and Creedy 2004; Dennis et al. 2024; Curry et al. 2019; Sockol 2015).

Although traditionally PND has been considered as depression in the first year after giving birth (National Institute for Health and Care Excellence 2020; American College of Obstetrics and Gynaecology 2023), it has been argued that the 12‐month cut‐off may be somewhat arbitrary. There is increasing evidence that the first 1001 days of life are the most critical, where consistency of healthcare provision is beneficial, particularly for new mothers with poor mental health struggling to bond with their babies (HM Government 2021). Furthermore, psychiatric‐related maternal deaths occur more frequently in the latter half of the first postpartum year (Knight et al. 2023), with suicide risk peaking towards the end of the first postpartum year (Thornton et al. 2013; Grigoriadis et al. 2017), which indicates that PND often does not resolve at 12 months postpartum.

In light of the evidence, in 2019 NHS England secured government resource to ensure that by 2023/2024 an additional 24 000 women per year with moderate to severe mental illness (including depression) would have access to specialist PMH care from preconception to 24 months after birth, including the availability of specialist PMH community care, improved access to evidence‐based psychological support and mental health checks for partners of women accessing PMH services (NHS England 2019). Previously, only women with severe mental illness would have access to PMH services and would be transferred to adult psychiatric or primary care general practitioner (GP) services at 12 months postpartum. Despite the extension in service in England, the numbers of women eligible for these extended services are currently unknown. Consequently, it is vital to understand the prevalence of depression in the second postpartum year to ensure the service can meet demand.

Most literature has focused on the prevalence of PND in the first postpartum year, with a recent systematic review of 565 studies estimating that 17.2% of mothers experience PND at some point during this time (Wang et al. 2021). This falls within previous estimates of 13.0%–19.2% (Shorey et al. 2018; Gavin et al. 2011; O'Hara and Swain 1996). Little is known, however, about the prevalence of PND after the first year. Two previous systematic reviews have found only small numbers of studies with prevalence estimates beyond the first year (Goodman 2004; Vliegen et al. 2014). One in 2004 looked at the prevalence of postpartum depression from 6 months to 2.5 years postpartum (Goodman 2004); however, only five studies were included that assessed PND beyond the first 12 months, with an estimated prevalence range for the second postpartum year of 13%–30% (Goodman 2004). A subsequent review of longitudinal studies found five studies with a prevalence range of 13%–36% in the second year (Vliegen et al. 2014). These are wide ranges, and an initial scope of the literature found that several studies not included in these reviews reported on PND beyond the first year, indicating that a more precise estimate would be possible.

The objective of this systematic review was to examine and evaluate the current global literature to obtain the best estimate of the prevalence of PND in women in the second postpartum year and beyond.

## 2 Methods

### 2.1 Study Design

This study is a systematic review and meta‐analysis, undertaken using the Preferred Reporting Items for Systematic Reviews and Meta‐Analysis (PRISMA) guidelines (Moher et al. 2009) (Data S1). The systematic review protocol was written prior to conducting the search.

### 2.2 Study Selection and Analysis

In January 2021, a systematic search of the current literature in PubMed, Embase, Web of Science, CINAHL and PsychINFO electronic databases, using search terms (Data S2), was conducted. To identify any additional studies, references of relevant studies were manually searched. Searches were updated in February 2024. Studies that assessed PND and provided a point prevalence using a validated screening tool or clinical diagnosis at least once beyond the first 12 months were included if they used an observational (cohort and cross‐sectional) design or provided data on, control groups from randomised controlled trials and quasi‐experimental trials. Studies that only included women who were already depressed or had elevated scores at baseline were excluded from the prevalence estimate. Although the focus of the review was to determine the prevalence of PND in the second year, given the negative long‐term outcomes associated with PND, looking beyond the second postpartum year was also deemed appropriate. Studies where data were not available in an appropriate format were excluded: data as mean scores, overall prevalence with no clear distinction between the first postnatal period and subsequent years, or insufficient information or as part of a secondary analysis.

**FIGURE 1:**
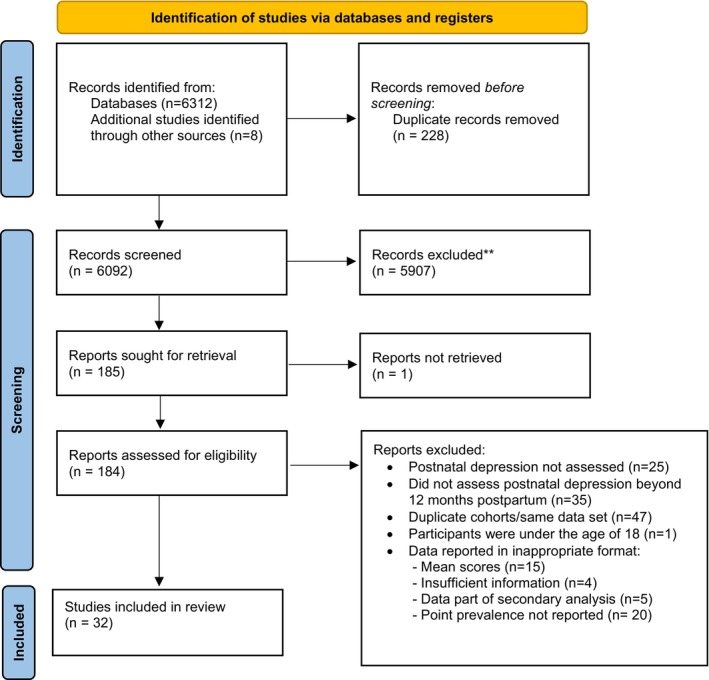
PRISMA flow diagram.

Initial papers generated from the databases were imported into Endnote referencing software and screened for duplicates (Figure 1). The remaining papers were then imported into Rayyan systematic review software. The titles and abstracts were screened independently by two authors (EH and KN), guided by the inclusion/exclusion eligibility criteria (Data S3). Results from the title and abstract screen were discussed to identify any inconsistencies, and any studies that did not meet the eligibility criteria were excluded. Studies still eligible were screened in full by EH and KN independently to determine inclusion in the review. Multiple papers reporting the same dataset were compared, and the most relevant paper was selected for data selection. Included studies were independently assessed for data extraction by the two authors (EH, KN).

### 2.3 Data Extraction

Data were extracted using a data extraction form, which was piloted on the first 10 studies to make any necessary amendments. Once data extraction was complete, two authors (EH, KN) discussed findings and checked for consistency. A third author (EJ) was contacted if issues were not resolved. Data on study characteristics including author, study population, study design, country, assessment tool and assessment periods were extracted from each study. Data regarding the prevalence of PND at reported time periods were extracted for synthesis.

### 2.4 Risk of Bias

The methodological quality of included cohort and cross‐sectional studies were assessed using Cochrane's ROBINS‐I tool for non‐randomised studies (Sterne et al. 2016). A modified version was used, with items relating to bias of intervention omitted due to the study design of this systematic review. When data were extracted from control groups in studies that included a randomisation process, the methodological quality was assessed using Cochrane's RoB 2 tool for randomised trials (Sterne et al. 2019). Studies were given rating of ‘low, moderate, serious, critical or not reported’ for each item and then an overall score based on these judgements. Independent quality assessment scoring was carried out by EH and KN and discussed to produce a final assessment.

### 2.5 Analysis

Data from studies reporting point prevalence of PND were pooled in a meta‐analysis to obtain estimates for PND in the first year (0–12 months), second year (13–24 months) and third year or later (> 25 months) postpartum. In the case where studies reported multiple prevalence estimates within the time period, the largest estimate was used for analysis as PND is already likely to be underreported (Chew‐Graham et al. 2009; Shafiei et al. 2015; Coates et al. 2014): both could not be given as this would have overrepresented that cohort within the meta‐analysis.

Prevalence information was either extracted directly from the studies or calculated using reported percentages and sample sizes for studies that did not report these statistics directly. Prevalence estimates across groups of studies were meta‐analysed using the Freeman–Tukey double Arcsine transformation to stabilise the variances. Forest plots with pooled estimates and corresponding 95% binomial exact confidence intervals (CIs) and prediction intervals (PIs) were produced. As recommended for prevalence meta‐analyses, we presented PIs alongside CIs for the pooled results to provide information on the expected range of results (Rücker et al. 2008; Inthout et al. 2016). All statistical analyses were conducted using Stata software, version 16.1.

Studies included in the meta‐analysis were further assessed to consider the continuation of PND in cases from the first postpartum year relative to new‐onset cases and for PND severity categorisation. Sub‐group analysis investigated differences according to assessment tools (screening tools or diagnostic interview).

### 2.6 Patient and Public Involvement

Four public contributors met with the research leads to discuss the study findings and their implications, and co‐developed with the research team key messages from their perspectives (see the Acknowledgements section).

### 2.7 Ethical Approval and Consent to Participate

This is a systematic review and meta‐analysis and did not require ethical approval.

## 3 Results

### 3.1 Study Selection

**FIGURE 2:**
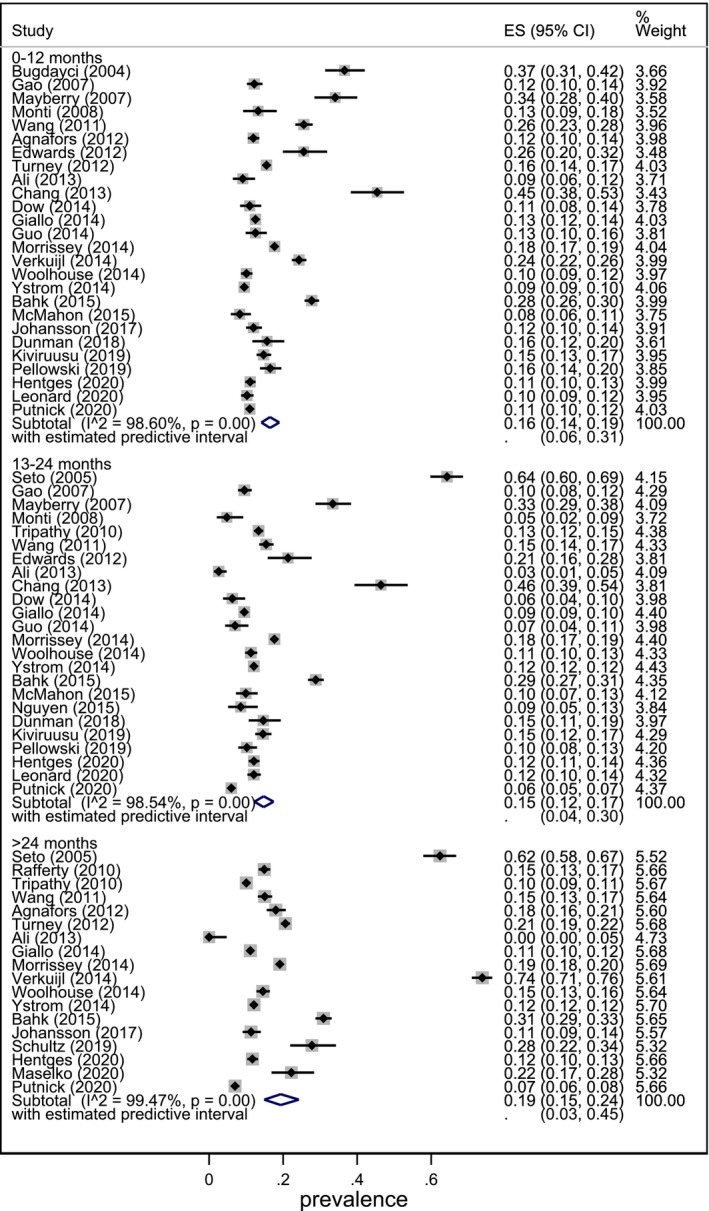
Meta‐analysis of pooled prevalence in the first year (0–12 months), second year (13–24 months) and after this (> 24 months) postpartum.

The initial literature search identified 6320 papers. After removing duplicate studies and undertaking the screening of titles and abstracts, 184 full texts were reviewed. Of these, 32 studies reported point prevalence of PND across the assessment periods of the first postpartum year (0–12 months), the second postpartum year (13–24 months) or after the second postpartum year (> 24 months) (Figure 2).

### 3.2 Description of Studies

Of the 32 included studies, 27 were cohort studies, 3 were cross‐sectional studies, 1 was a study of a control group from a randomised controlled trial and 1 was a quasi‐experimental study (Table 1). The number of included participants was 57 210. The studies were published over a 16‐year period from 2004 to 2020, in 18 countries, including 5 from Europe, 10 from North America, 1 from South America, 4 from Africa, 8 from Asia and 4 from Australia and New Zealand. There was a large variation in study sizes, ranging from 42 (Edwards et al. 2012) to 19 189 (Ystrom et al. 2015) participants. Only two studies had fewer than 100 participants (Edwards et al. 2012; Ali et al. 2013), with more than half of the included studies having a study sample of over 1000 participants.

**TABLE 1:** Description of 32 studies that reported PND point prevalence and were included in meta‐analysis.

Author (year)	Location	Study type	Screening tool	Outcome definition	Sample size at final follow‐up	Postpartum assessment time (prevalence estimate)
Agnafors et al. (2013)	Sweden	Cohort	Edinburgh Postnatal Depression Scale (EPDS) and SCL‐25	EPDS ≥ 10, SCL‐25 1.75	893	3 months (12%), 12 years (18.2%)
Ali et al. (2013)	Pakistan	Quasi‐experimental	Aga Khan University Anxiety and Depression Scale (AKUADS)	AKUADS > 19	76	1 month (4.8%), 2 months (4.7%), 6 months (5.7%), 12 months (9.2%), 18 months (2.7%), 2 years (6.1%), 3 years (0%)
Bahk et al. (2015)	Korea	Cohort	Kessler 6 (K6)	K6 ≥ 14	1754	4 months (27.7%), 12 months (26.6%), 2 years (28.8%), 3 years (30.9%)
Bugdayci et al. (2004)	Turkey	Cross‐sectional	EPDS	EPDS ≥ 13	515	0–2 months (29.0%), 3–6 months (36.6%), 7–12 months (36.0%), > 13 months (47.7%)
Chang et al. (2018)	Taiwan	Cohort	Center for Epidemiologic Studies Depression Scale (CES‐D)	CES‐D ≥ 16	196	4–6 weeks (54.1%), 3 months (43.9%), 6 months (42.4%), 12 months (45.4%), 2 years (46.4%)
Dow et al. (2014)	Malawi	Cohort	EPDS	EPDS ≥ 12	276	10–14 weeks (11.0%), 6 months (7.7%), 9 months (10.5%), 12 months (7.4%), 15 months (6.3%), 18 months (2.9%)
Duman et al. (2018)	Turkey	Cohort	EPDS	EPDS ≥ 13	280	4 months (13.7%), 13 months (15.6%), 20 months (14.8%)
Edwards et al. (2012)	USA	Cohort	CES‐D	CES‐D > 16	42	4 months (25.6%), 12 months (22.4%), 2 years (21.3%)
Gao et al. (2007)	New Zealand	Cohort	EPDS and General Health Questionnaire‐12 (GHQ‐12)	EPDS > 12, GHQ‐12	1144	6 weeks (16.1%), 12 months (12.2%), 2 years (9.5%)
Giallo et al. (2014)	Australia	Cohort	K6	K6 8–12 mild, > 13 moderate/severe	4242	3–12 months (12.8%), 2–3 years (9.6%), 4–5 years (11.1%), 6–7 years (10.1%)
Guo et al. (2014)	Ghana and Côte d'Ivoire	Cohort	Patient Health Questionnaire‐9 (PHQ‐9)	PHQ‐9 ≥ 10	309	3 months (10.9%), 12 months (12.5%), 2 years (7.0%)
Hentges et al. (2020)	Canada	Cohort	EPDS and CES‐D	EPDS > 10, CES‐D > 16	1992	4 months (10.2%), 12 months (11.1%), 2 years (12.1%), 3 years (11.7%)
Johansson et al. (2017)	Sweden	Cohort	EPDS	EPDS ≥ 12	700	3 months (12.0%), 25 months (11.3%)
Kiviruusu et al. (2020)	Finland	Cohort	CES‐D	CES‐D ≥ 10	1038	3 months (10.5%), 8 months (14.7%), 2 years (14.5%)
Leonard et al. (2020)	USA	Cohort	EPDS	EPDS > 10	1316	1 month (9.5%), 6 months (8.3%), 12 months (10.2%), 18 months (10.1%), 2 years (12.1%)
Maselko et al. (2020)	Pakistan	Cohort	SCID	Clinical judgement	467	3 years (9.0%)
Mayberry et al. (2007)	USA	Cross‐sectional	EPDS	EPDS > 10 mild, > 10 < 13 moderate, > 13 severe	1359	0–6 months (34.1%), 7–12 months (31.2%), 13–18 months (28.1%), 19–24 months (33.5%)
Mcmahon et al. (2015)	Australia	Cohort	Mini International Neuropsychiatric Interview (MINI) and EPDS	EPDS ≥ 13	434	4 months (8.3%), 2 years (9.9%)
Monti et al. (2008)	Italy	Cohort	EPDS	EPDS ≥ 13	167	3 months (13.2%), 9 months (10.6%), 18 months (8.9%)
Morrissey and Dagher (2014)	USA	Cohort	CES‐D and Composite International Diagnostic Interview (CIDI‐SF)	CES‐D ≥ 9 mod, > 15 severe	4850	9 months (17.7%), 2 years (17.6%), 4 years (19.1%), 5 years (16.5%)
Nguyen et al. (2015)	Vietnam	Cohort	Structured clinical interviews	Psychiatrist/DSM‐IV	211	15 months (8.5%)
Pellowski et al. (2019)	South Africa	Cohort	EPDS	EPDS > 13 mod	831	10 weeks (17.1%), 6 months (14.5%), 12 months (16.5%), 18 months (10.2%)
Putnick et al. (2020)	USA	Cohort	EPDS (5‐item)	EPDS ≥ 7	2131	4 months (11.0%), 12 months (8.0%), 2 years (6.0%), 3 years (7.0%)
Rafferty et al. (2010)	USA	Cohort	CES‐D	CES‐D ≥ 15	2040	3 years (14.9%)
Schultz et al. (2020)	Brazil	Cross‐sectional	BDI	BDI ≥ 12 moderate	216	4–5 years (27.8%)
Seto et al. (2005)	USA	Cohort	CES‐D	CES‐D ≥ 16	476	18 months (64.3%), 3 years (62.4%), 6 years (56.9%), 10 years (60.1%)
Tripathy et al. (2010)	India	RCT‐controls	Kessler 10 (K10)	K10 > 16 moderate/severe	2963	2 years (14.0%), 3 years (11.0%)
Turney (2012)	USA	Cohort	CIDI‐SF	Clinical judgement	3625	12 months (15.5%), 3 years (20.6%), 5 years (17.0%), 9 years (17.4%)
Verkuijl et al. (2014)	South Africa	Cohort	Pitt depression inventory and CES‐D	Pitts ≥ 20, CES‐D ≥ 16	1012	6 months (24.0%), 10 years (74.0%)
Wang et al. (2011)	USA	Cohort	CES‐D	CES‐D ≥ 16	1364	1 month (25.6%), 6 months (16.3%), 15 months (15.4%), 2 years (15.0%), 3 years (15.0%)
Woolhouse et al. (2015)	Australia	Cohort	EPDS	EPDS ≥ 13	1102	3 months (8.1%), 6 months (10.1%), 12 months (9.5%), 18 months (11.3%), 4 years (14.5%)
Ystrom et al. (2015)	Norway	Cohort	Symptom Check List‐8 (SCL‐8)	> 1.75	19 189	6 months (9.5%), 18 months (12.1%), 3 years (12.5%), 5 years (8.5%)

The 32 studies included in the meta‐analysis covered a follow‐up period of up to 12 years postpartum. Most studies reported the prevalence of depression at multiple time points in the postpartum period (Table 1): 26 studies reported the prevalence in the first postpartum year (0–12 months), 25 studies reported the prevalence in the second postpartum year (13–24 months) and 18 studies reported the prevalence beyond the second postpartum year (> 24 months).

Across all assessment periods, PND was most often assessed using screening tools. Of the screening tools, 10 studies used the Edinburgh Postnatal Depression Scale (EPDS) (Duman et al. 2018; Johansson et al. 2017; Leonard et al. 2020; Mayberry et al. 2007; Monti et al. 2008; Pellowski et al. 2019; Putnick et al. 2020; Woolhouse et al. 2015; Bugdayci et al. 2004; Dow et al. 2014), six used the Center for Epidemiologic Studies Depression Scale (CES‐D) (Chang et al. 2018; Edwards et al. 2012; Kiviruusu et al. 2020; Rafferty et al. 2010; Seto et al. 2005; Wang et al. 2011), 7 studies used a different tool (Ali et al. 2013; Bahk et al. 2015; Giallo et al. 2014; Guo et al. 2014; Schultz et al. 2020; Ystrom et al. 2015; Tripathy et al. 2010) and 4 studies used a combination of screening tools (Agnafors et al. 2013; Gao et al. 2007; Hentges et al. 2020; Verkuijl et al. 2014).

Three studies used only clinical interviews to estimate PND prevalence: one at 12 months and at 3, 5 and 9 years (Turney 2012), one at 15 months (Nguyen et al. 2015) and one at 3 years (Maselko et al. 2020). Two studies used a combination of interview and screening tool (Mcmahon et al. 2015; Morrissey and Dagher 2014); they both used the screening tool to calculate their point prevalence estimates. We were unable, therefore, to undertake our sub‐group analysis of making a comparison of differences in PND prevalence according to assessment tools.

### 3.3 Risk of Bias

The risk of bias was low–moderate for most studies, across the four risk of bias domains, with no studies earning a critical rating. Of the 32 studies, 5 were given a rating of ‘low’ risk of bias, 25 were given ‘moderate’ and 1 was given ‘serious’ (Tables 2 and 3). A serious risk is judged to have one or more important problems but is not a ‘critical’ risk and therefore should still be included in a synthesis (Sterne et al. 2022).

**TABLE 2:** Risk of bias I.

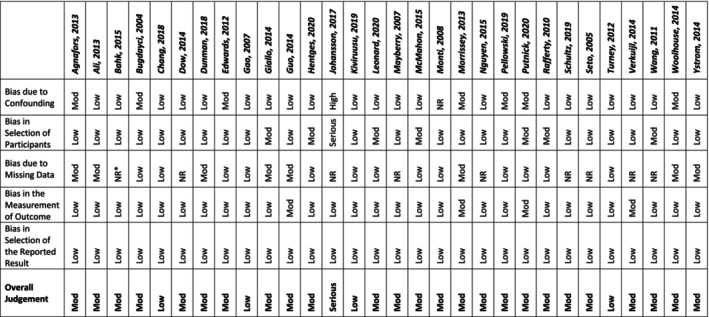

**TABLE 3:** Risk of bias II.

			Maselko et al. (2020)	Tripathy et al. (2010)
Bias due to randomisation process			Low	Low
Bias due to deviations from the intended interventions			Low	Low
Bias due to missing data			Low	NR
Bias in the measurement of outcome			Low	Low
Bias in selection of the reported result			Low	Low
Overall judgement			Low	Mod

### 3.4 Prevalence

Results showed that the overall pooled prevalence of PND in the second postpartum year (13–24 months) was 15% (95% CI 12%, 17%; 95% PI, 4%, 30%) and was not significantly different from the pooled prevalence estimated during the first postpartum year (0–12 months) (16%; 95% CI 14%, 19%; PI 6%, 31%). Similar prevalence also continues into the third postpartum year and after (> 24 months) (19%, 95% CI 15%, 24%, PI 3%,45%). There was considerable heterogeneity in the pooled estimate of prevalence across all postpartum years: first year (*I* ^2^ 98.6%), second year (*I* ^2^ 98.54%) and third year (*I* ^2^ 99.47%).

### 3.5 Continuation of PND From Year 1 and New Onset

Of the 32 studies reporting the prevalence of PND, five also subcategorised women with PND into those with depression that continued from the first postpartum year and those with PND that started after the first postpartum year (Mcmahon et al. 2015; Monti et al. 2008; Turney 2012; Wang et al. 2011; Woolhouse et al. 2014) (Data S4).

Two studies found high levels of continued cases, with the proportion of women who had reported PND during the first year postpartum also reporting PND at 3 and 4 years being 52.1% and 42.8% (Turney 2012; Woolhouse et al. 2015). One study reported lower levels of continued cases, with 25.0% of the women with PND in the first postpartum year still reporting depression at 2 years (Mcmahon et al. 2015).

Two studies specifically reported on new‐onset cases of PND after the first year postpartum, with the prevalence of new cases out of the total sample of women found to be 4.8% and 7.4% at 18 and 24 months, respectively (Monti et al. 2008; Wang et al. 2011).

### 3.6 Severity of PND

Of the 32 studies reporting the prevalence of PND, five studies subcategorised by severity (mild, moderate and severe) (Giallo et al. 2014; Mayberry et al. 2007; Morrissey and Dagher 2014; Schultz et al. 2020; Tripathy et al. 2010) (Data S5).

For the second year postpartum, two studies reported the proportion of women experiencing moderate to severe depression as 14.0% and 20.4%, respectively (Tripathy et al. 2010; Mayberry et al. 2007). The prevalence of moderate to severe depression seemed to remain fairly stable across the assessment periods. One study reported a similar prevalence of moderate to severe depression in the first year (23.1%) as in the second year (20.4%) postpartum (Mayberry et al. 2007). Tripathy et al. (2010) also reported a similar proportion of women with moderate to severe depression in the second (14%) and the third year postpartum (11.0%). Another study reported the prevalence of moderate to severe depression in the fourth postpartum year across the sample to be 16.6% (Schultz et al. 2020).

Two studies also found that the proportion of women experiencing severe depression remained the same throughout the study follow‐up period, 1–5 and 1–7 years, respectively (Morrissey and Dagher 2014; Giallo et al. 2014). Of all women with PND, the proportion experiencing severe depression ranged between 2.2% and 2.8% (Giallo et al. 2014) and 5% and 8.0% (Morrissey and Dagher 2014).

## 4 Discussion

This systematic review is the only review to comprehensively quantify the proportion of women with depression after the first 12 months postpartum. Out of 32 studies included in the meta‐analysis, 24 with prevalence data for the second postpartum year (15%, 95% CI 12%, 17%) indicated that prevalence was similar to that of the first year (16%, 95% CI 14%, 19%). The results for the third or subsequent postpartum years (> 24 months) (19%, 95% CI 15%, 24%) suggest that longer term extensions may also be beneficial. Whether or not depression at this time would still be considered postpartum, however, is open to question.

Our study finding of high prevalence of depression in the second postpartum year has important implications for treatment to improve outcomes for both mothers and their offspring. Psychosocial (e.g., peer support, non‐directive counselling) and psychological (e.g., cognitive behavioural therapy and interpersonal psychotherapy) interventions are known to be effective treatments for PND in the first year (Howard et al. 2014; Dennis and Hodnett 2007), with some evidence on the effectiveness of antidepressant medication (Brown et al. 2021). Given the differences in context when treating PND (such parental caring responsibilities, breastfeeding and sleep disturbances), specific psychological and psychosocial interventions that are effective in treating PND in the first postpartum year would likely benefit women in the second postpartum year due to the similar challenges that women face.

Emphasis should also be placed on reducing the risk of adverse outcomes among offspring, as treating PND does not always improve outcomes for maternal–infant bonding (Stein et al. 2014). Whilst genetic, other biological and environmental mechanisms by which risks to offspring occur are complex (Stein et al. 2014), the adverse effects of perinatal mental illness on infants and children, including adverse psychosocial development and a higher risk of long‐term morbidity, have been documented (Aktar et al. 2019; Erickson et al. 2019; Jacques et al. 2019). Hence, recognising the high prevalence in the second year is important in the provision of care and improvement of long‐term outcomes for infants.

A strength of this review is the large number of studies included: the only previous literature reviews of postpartum depression after the first year (Goodman 2004; Vliegen et al. 2014) were each limited to five studies of PND beyond the first 12 months and did not meta‐analyse the studies. The reported prevalence ranges from included studies were from 13.0% to 30.6% (Goodman 2004) and 13%–46% (Vliegen et al. 2014), and although these are wide ranges, they do encompass the more precise estimate of 15.0% from our review. The searches were updated in February 2024, and one additional study was eligible for inclusion, although it only provides data on the prevalence of PND at 1, 3 and 5 years and therefore is unlikely to affect the main findings of the review of PND in the second postpartum year (Pineros‐Leano et al. 2021). A population‐based study of 547 747 children in the United Kingdom showed that the prevalence of maternal depression among children aged 0–2 years was 17.6% (17.4%–17.7%). Whilst this study did not examine prevalence in the second year separately from the first, the prevalence estimate is very similar to our estimate, further emphasising the need within the first 2 years. Another study of 3290 psychiatric admissions in Scotland found that weekly psychiatric admission rates were higher in the early postpartum period 0–7 weeks (46.2) and remained elevated in the late postpartum period (6 weeks–2 years) (18.8) compared to pre‐pregnancy (13.3) and during pregnancy (7.9). The most common reason for admission in the late postpartum period was non‐psychotic depression. There have been several systematic reviews of PND only during the first postpartum year, the most recent in 2021, which reported a prevalence of 17.2 (95% CI 16.0–18.5) (Wang et al. 2021), similar to earlier review estimates of 13%–19.2% (Shorey et al. 2018) (Gavin et al. 2011; O'Hara and Swain 1996). These review estimates only include women with PND in the first 12 months postpartum, which could potentially be a different group of women than those included in the longer term studies. However, the prevalence estimates from these reviews are similar to those found for the first 12 months in our review.

The meta‐analysis showed considerable heterogeneity, with the I^2^ statistic above 90.0%. This could be due to various factors, including the use of different screening tools, variable cut‐off scores for positive results, studies from a variety of countries and regions, and different populations. This could be considered a weakness; however, there is evidence that heterogeneity can be overestimated when summarising studies with large sample sizes and increases with the number of patients included. A higher *I* ^2^ is therefore expected when pooling estimates from different times and locations (Rücker et al. 2008). The *I* ^2^ does not directly relate to the distribution of effects, and a high *I* ^2^ value does not always indicate important heterogeneity. As recommended for prevalence meta‐analyses, we presented PIs alongside CIs for the pooled results to provide information on the expected range of results (Rücker et al. 2008). The original protocol is available from the authors on request.

Secondary analyses considered the prevalence of depression continuing into the second year and severity; however, these were specifically examined in only a few studies. Those that did examine continuation prevalence suggested that up to 50% of women who had PND in the first year still experienced PND beyond the first 12 months postpartum. This suggests that the women requiring PMH services will include both those who are experiencing continued depression and those with post‐year 1 onset depression.

Current UK NHS PMH services, in addition to being extended to include the second year, have been modified to include women who experience moderate and severe depression (mild depression is treated within primary care). Again, only a small number of studies in this review sub‐divided depression according to severity, but those that did suggested that moderate and severe depression remained similarly prevalent during the first and second postpartum years. These questions would need to be investigated using other research methods, for example, cohort studies.

Screening tools that rely on self‐reported accounts of depression are an extremely accessible method of assessing PND and hence their frequent utilisation in much of the research literature. This provides us with more data than purely from clinical diagnosis, enabling us to calculate a more accurate estimate for PND prevalence. However, a positive screen using assessment tools is not synonymous with a clinical diagnosis (National Institute for Health and Clinical Excellence 2014; Milgrom et al. 2011). Estimating the prevalence of PND from screening tools alone, therefore, is likely to have overrepresented the size of the proportion of women with depression who will need specialist care; however, it remains the best estimate currently available.

## 5 Conclusion

This review of global data showed that the prevalence of depression in the second year postpartum, at 15%, is similar to the 16% found in the first year and provides the most comprehensive estimate of PND occurring beyond the first 12 months postpartum to date. Further research should consider the prevalence of other mental illnesses in the second postpartum year, as women may also benefit from specialist treatments offered by PMH services. Determining the prevalence of depression by its severity in the second postpartum year and determining for how long psychological, psychosocial and pharmacological treatments offered within specialist PMH services are beneficial in improving maternal and child outcomes are essential so that finite resources can be managed appropriately.

## 6 Relevance for Clinical Practice

Given the poor long‐term maternal and child outcomes associated with PND, healthcare professionals should be aware of the high prevalence of depression within the second year and consider referral and treatment within specialist PMH services were available to ensure optimal outcomes for women and their babies. From the perspective of those with lived experience, health professionals should also be cognisant that it may be challenging for women to talk about their symptoms due to the stigma associated with PND and that there may be many women who are struggling but not accessing support from health services and may have been suffering for some time. Women should be routinely made aware of symptoms of PND and those experiencing depression in the first postpartum year should be aware of the provision of specialist services in the second year in countries where this service is available. In terms of service provision, this review supports NHS England's decision to extend specialist PMH to 2 years, although longer term extensions may also be helpful. Countries with specialist PMH services, such as Australia, may also benefit from a similar extension of care.

## Author Contributions

C.M. and E.J.: conceptualisation, methodology, formal analysis, writing – original draft and editing. E.H.: methodology, data extraction, writing – original draft and editing. K.N.: data extraction formal analysis, writing – editing. A.C.: data extraction, writing – editing. J.J. and G.B.: conceptualisation, writing – editing. A.S.: data analysis, writing – editing. All authors read and approved the final manuscript.

## Ethics Statement

This is a systematic review and meta‐analysis and does not require ethical approval.

## Conflicts of Interest

All authors have completed the Unified Competing Interest form: C.M. and E.J. declare National Institute for Health research (NIHR) funding to University of Birmingham for various research projects, J.J. and G.B. declare NIHR funding to QMUL for the PAAM study, J.J. is academic secretary in the Royal College of Psychiatrists and G.B. was previous vice chair and now elected member of the Faculty of Perinatal Psychiatry at the Royal College of Psychiatrists and National Speciality for Perinatal Psychiatry with NHS England.

## Supporting information


Data S1.

Data S2.

Data S3.

Data S4.

Data S5.

## Data Availability

Data sharing is not applicable to this article as no new data were created or analyzed in this study.
