# Supplementary material for: Postnatal Depression Beyond 12 Months: A Systematic Review and Meta‐Analysis
Source: Int J Ment Health Nurs. 2025 Mar 7;34(2):e70018. doi: 10.1111/inm.70018 (PMC11889294; doi:10.1111/inm.70018)
Supplement: Supplementary file 2 — Data S2. [file INM-34-0-s003.pdf]

	**Search Terms**
**1**	PND or<br>Postnatal depression or<br>Post-natal depression or<br>Postpartum depression or<br>Post-partum depression or<br>Postnatal depressive symptoms or<br>Postpartum depressive symptoms or<br>Postnatal psychosis or<br>Postnatal puerperal or<br>Postnatal mood disorders or<br>Postpartum mood disorders or<br>Maternal depression or<br>Maternal depressive symptoms or<br>Depression after birth
**2**	After 12 months or<br>Beyond 12 months or<br>After a year or<br>Long term or<br>Longitudinal or<br>Persistent or<br>Continued or<br>Trajectories
**3**	Prevalence or<br>Incidence
**4**	1 and 2 and 3
**5**	Limit 4 to study design "observational study" or "cohort study" or "case-control study" or "cross-sectional study" or "Randomised controlled trial" or "RCT"
