# Supplementary material for: Postnatal Depression Beyond 12 Months: A Systematic Review and Meta‐Analysis
Source: Int J Ment Health Nurs. 2025 Mar 7;34(2):e70018. doi: 10.1111/inm.70018 (PMC11889294; doi:10.1111/inm.70018)
Supplement: Supplementary file 3 — Data S3. [file INM-34-0-s004.pdf]

Category	*Inclusion Criteria*	*Exclusion Criteria*
**Population**	• Women with postnatal depression beyond the first 12 months postpartum*	• Women <18 years at time of pregnancy<br>• No limit to the severity or duration of the participants symptoms<br>• Women with depression identified prior to or at baseline
**Interventions**	N/A**	N/A**
**Comparators**	N/A**	N/A**
**Outcome**	• Primary outcome of presence of depression or depressive symptoms in women more than one year after giving birth<br>• Severity and continuation of postnatal depression in these women in the same time period will also be looked for.<br>• Relevant studies must have assessed PND on at least one occasion beyond the first 12 months postpartum, using a validated assessment tool e.g. the Edinburgh Postnatal Depression Scale.	• Severity and continuation of postnatal depression will only be assessed in studies evaluating the primary outcome.<br>• Studies reporting outcomes in an inappropriate format***
**Study Design and Features**	• Observational studies, including cross-sectional, cohort or case-control studies, will be primarily searched for<br>• Randomised controlled trials will also be included if they have assessed prevalence of postnatal depression<br>• English language only<br>• Published before Feb 2021	• Case-studies excluded

* Presence of PND should be identified with either a clinical diagnosis or screened for likely depression using a validated assessment tool.

**No inclusion/exclusion criteria or search terms relating to interventions or comparators/control groups. This is because this SR is focused on prevalence/incidence and severity of disease, therefore there is no need for a comparator group or to assess for interventions.

***Inappropriate format was defined as studies that reported data as mean scores, overall prevalence with no clear distinction between first postnatal period and subsequent years, insufficient information or as part of a secondary analysis.
