# Supplementary material for: Postnatal Depression Beyond 12 Months: A Systematic Review and Meta‐Analysis
Source: Int J Ment Health Nurs. 2025 Mar 7;34(2):e70018. doi: 10.1111/inm.70018 (PMC11889294; doi:10.1111/inm.70018)
Supplement: Supplementary file 4 — Data S4. [file INM-34-0-s005.pdf]

Author, Year	Assessment Tool	Assessment Periods	Prevalence	Continued Cases
**Monti, 2008**	Edinburgh Postnatal Depression Scale	3 months<br>9 months<br>18 months	13.2%<br>10.6%<br>8.9%	New onsets: 13.2% at 3 months, 5.5% at 9 months, 4.8% at 18 months<br><br>1.2% showed persistent depression at all three assessments<br><br>1.2% depressed at both 9 and 18 months<br>1.8% depressed at both 3 and 18 months<br>3.0% depressed at both 3 and 9 months
**McMahon, 2015**	Mini International Neuropsychiatric Interview	4 months<br>2 years	8.3%<br>9.9%	25.0% of those depressed in the first four months (and 2.1% of the total sample) met the criteria for depression on both occasions
**Turney, 2012**	Composite International Diagnostic Interview – Short Form	1 year<br>3 years<br>5 years<br>9 years	15.6%<br>20.6%<br>17.0%<br>17.4%	52.0% of mothers depressed at 1 year also depressed at 3 years<br>43.0% depressed at 3 years also depressed at 5 years<br>44.0% depressed at 5 years also depressed at 9 years<br><br>31.0% reported intermittent depression (at 1 or 2 time points)<br>7.0% reported persistent depression (at 3 or 4 time points)
**Wang, 2011**	Center for Epidemiologic Studies Depression Scale	6 months<br>15 months<br>2 years<br>3 years	16.3%<br>15.4%<br>15.0%<br>15.0%	Depression status was further defined as early onset (occurring within 6 months after giving birth), late onset (occurring when the child was at least 24 months old), and chronic depression (depression lasting from early onset to late onset or beyond).<br>The prevalence of early onset, late onset, and chronic maternal depression was 32.2%, 7.4%, and 13.4%, respectively.
**Woolhouse, 2014**	Edinburgh Postnatal Depression Scale	3 months<br>6 months<br>12 months<br>18 months<br>4 years	8.1%<br>10.1%<br>9.5%<br>11.3%<br>14.5%	At 4 years, 59.5% had previously reported depressive symptoms (48.1% on at least one occasion in the first 12 months).<br>53.5% scored ≥13 on EPDS on one occasion<br>20.1% on two occasions<br>11.5% on three occasions<br>14.9% on four or more.<br><1.0% at every follow-up
