# Supplementary material for: Postnatal Depression Beyond 12 Months: A Systematic Review and Meta‐Analysis
Source: Int J Ment Health Nurs. 2025 Mar 7;34(2):e70018. doi: 10.1111/inm.70018 (PMC11889294; doi:10.1111/inm.70018)
Supplement: Supplementary file 5 — Data S5. [file INM-34-0-s001.pdf]

Author, Year	Assessment Tool	Assessment Periods	Prevalence	Severity Definitions	Severity Prevalence
**Giallo, 2014**	Kessler-6 (K6)	12 months<br>2-3 years<br>4-5 years<br>6-7 years	12.80%<br>9.60%<br>10.10%<br>11.40%	Reported as either in the symptomatic range (K6 ≥8) or clinical range (>13)	Clinical range:<br>2.80%<br>2.20%<br>2.50%<br>2.80%
**Mayberry, 2007**	Edinburgh Postnatal Depression Scale (EPDS)	0-6 months<br>7-12 months<br>13-18 months<br>19-24 months	34.10%<br>31.20%<br>28.10%<br>33.50%	Reported as mild (EPDS 10-12) or moderately-severely (EPDS ≥13) depressed	Mild, mod-severe:<br>11.00%, 23.10%<br>15.10%, 16.10%<br>11.00%, 17.10%<br>13.10%, 20.40%
**Morrissey, 2013**	Center for Epidemiologic Studies Depression Scale (CES-D) and Composite International Diagnostic Interview (CIDI)	9 months<br>2 years<br>4 years<br>5.5 years	17.68%<br>17.64%<br>19.11%<br>16.54%	Reported overall proportions of women experiencing moderate (CES-D ≥9) or severe (CES-D >15)	Overall: 17-19% of mothers considered as having mod or severe symptoms, with 5-8% of these being severe
**Schultz, 2020**	Beck Depression Inventory (BDI-II)	4-5 years	16.70%	Reported as minimal (BDI 0-11), mild (BDI 12-19), moderate (BDI 20-35) or severe (36-63) depression	Min, mild, mod-severe:<br>52.30%, 14.40%, 16.60%
**Tripathy, 2010**	Kessler-10 (K10)	2 years<br>3 years	14.00%<br>11.00%	Reported as no/mild (K10 10-15), moderate (K10 16-30) or severe (K10 31-50) depression	Mild, mod-severe:<br>87.00%, 14.00%<br>90.00%, 11.00%
